# Nociceptive stimulation induces expression of Arc/Arg3.1 in the spinal cord with a preference for neurons containing enkephalin

**DOI:** 10.1186/1744-8069-6-43

**Published:** 2010-07-23

**Authors:** Mehdi Hossaini, Joost LM Jongen, Karla Biesheuvel, Dietmar Kuhl, Jan C Holstege

**Affiliations:** 1 Dept. of Neuroscience, Erasmus University Medical Center, PO Box 2040, 3000 CA, Rotterdam, The Netherlands; 2 Dept. of Neurology, Erasmus University Medical Center, PO Box 2040, 3000 CA, Rotterdam, The Netherlands; 3 Institute for Molecular and Cellular Cognition, Center for Molecular Neurobiology (ZMNH), University Medical Center Hamburg-Eppendorf, Falkenried 94, 20251 Hamburg

## Abstract

**Background:**

In pain processing, long term synaptic changes play an important role, especially during chronic pain. The immediate early gene Arc/Arg3.1 has been widely implicated in mediating long-term plasticity in telencephalic regions, such as the hippocampus and cortex. Accordingly, Arc/Arg3.1 knockout (KO) mice show a deficit in long-term memory consolidation. Here, we identify expression of Arc/Arg3.1 in the rat spinal cord using immunohistochemistry and in situ hybridization following pain stimuli.

**Results:**

We found that Arc/Arg3.1 is not present in naïve or vehicle treated animals, and is *de novo *expressed in dorsal horn neurons after nociceptive stimulation. Expression of Arc/Arg3.1 was induced in an intensity dependent manner in neurons that were located in laminae I (14%) and II (85%) of the spinal dorsal horn. Intrathecal injection of brain derived neurotrophic factor (BDNF) also induced expression of Arc/Arg3.1. Furthermore, 90% of Arc/Arg3.1 expressing neurons also contained the activity marker c-Fos, which was expressed more abundantly. Preproenkephalin mRNA was found in the majority (68%) of the Arc/Arg3.1 expressing neurons, while NK-1 was found in only 19% and GAD67 mRNA in 3.6%. Finally, pain behavior in Arc/Arg3.1 KO mice was not significantly different from their wild type littermates after application of formalin or after induction of chronic inflammatory pain.

**Conclusions:**

We conclude that Arc/Arg3.1 is preferentially expressed in spinal enkephalinergic neurons after nociceptive stimulation. Therefore, our data suggest that Arc/Arg3.1 dependent long term synaptic changes in spinal pain transmission are a feature of anti-nociceptive, i.e. enkephalinergic, rather than pro-nociceptive neurons.

## Background

The experience of pain is usually initiated by the activation of nociceptors, which are the peripheral terminations of nociceptive ganglion neurons. The central projections of these neurons enter the dorsal horn of the spinal cord to terminate on second order neurons [1]. After strong nociceptive stimulation these neurons may show an enhanced responsiveness to afferent inputs, which may last for several hours [2-4]. The mechanism underlying this enhanced responsiveness is similar to that of long-term potentiation (LTP) [5], which is a form of activity dependent plasticity that has been investigated extensively in other parts of the CNS, especially in the hippocampus [6]. Another form of activity dependent plasticity is long-term depression (LTD), a state of decreased sensitivity of neurons. Whether LTP or LTD is produced in the spinal nociceptive system depends on many variables, including the type of activity in nociceptive afferents [2]. For long term changes to become persistent it is essential that activity regulated genes, including immediate early genes (IEG), orchestrate a cascade of transcriptions and subsequent protein synthesis [7]. The first IEG that was found to be strongly increased in spinal neurons after a nociceptive stimulus is c-Fos [8]. This IEG is now widely used for the identification of activated nociceptive neurons [9]. Other IEGs that have been implicated in plastic changes are c-Jun, Jun-d, Krox 24 and Homer 1a [10,11]. Recently it has become clear that in cortex, hippocampus and other higher brain centers, the IEG named Arc/Arg3.1 (activity regulated cytoskeleton-associated protein/activity regulated gene 3.1) plays a crucial role in activity dependent synaptic plasticity [12]. Moreover, Arc/Arg3.1 is critically involved in processes essential for synaptic structural rearrangement such as LTP, LTD and homeostatic scaling of AMPA receptors [13,14]. These mechanisms are also essential in spinal processing [15], and dysfunctional forms of activity dependent plasticity such as LTP and LTD that lead to persistent changes in neuronal sensitivity, may underlie chronic pain disorders [16]. Therefore, in this study we set out to investigate the role of Arc/Arg3.1 in nociceptive processing in the spinal cord.

Our findings show that Arc/Arg3.1 is not expressed at detectable levels in naïve spinal cord. However, after peripheral nociceptive stimulation we found *de novo *expression of Arc/Arg3.1 in a limited number of neurons in the superficial dorsal horn, depending on the type of stimulus. Further, Arc/Arg3.1 is predominantly expressed in spinal interneurons located in lamina II and many of these neurons also contain the opioid neurotransmitter enkephalin. Finally, we found that the pain behavior in Arc/Arg3.1 knockout (KO) mice after nociceptive stimuli was not significantly different from their wild type (WT) littermates.

## Results

### General observations

**Figure 1:**
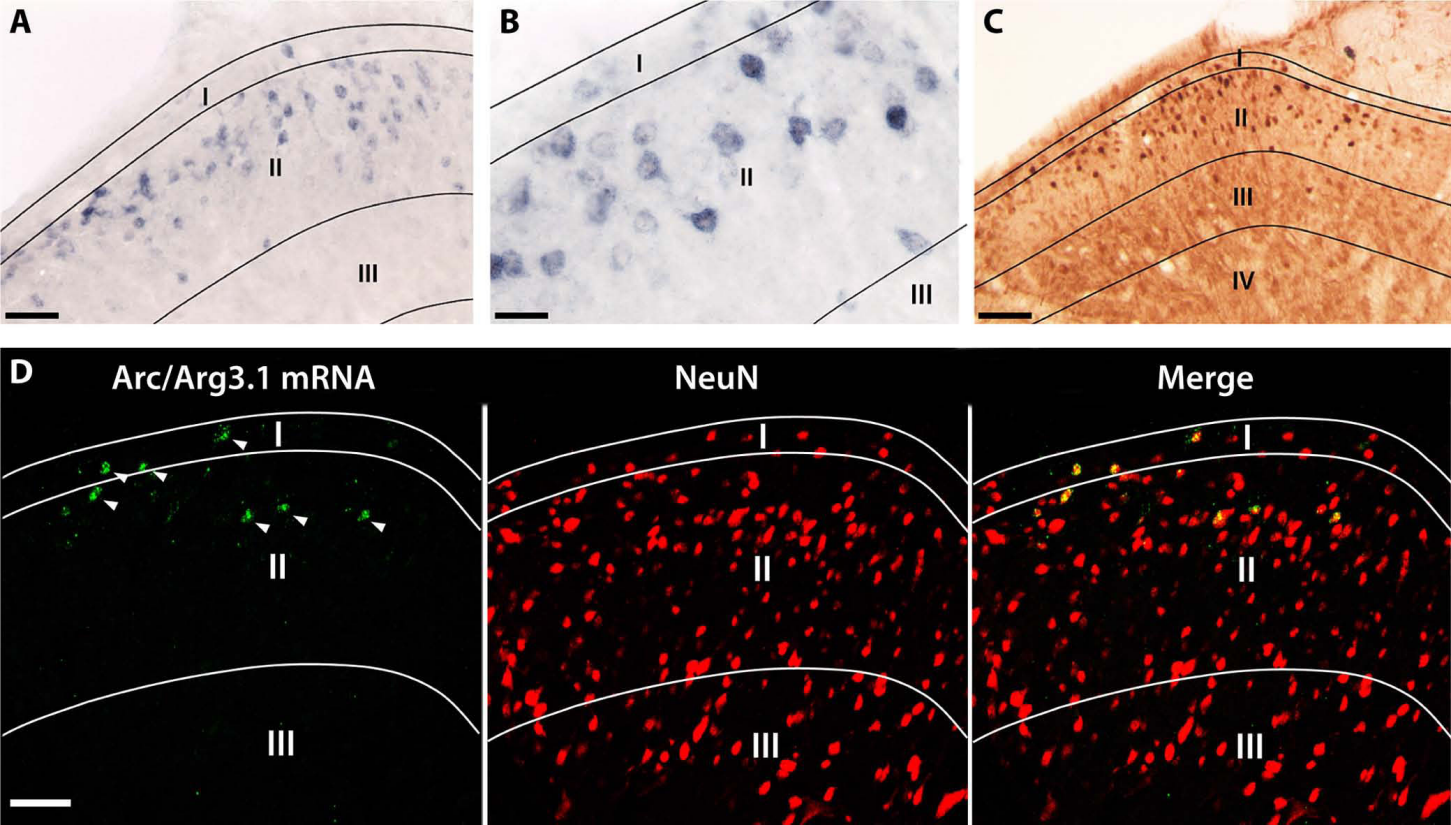
**Arc/Arg3.1 is only expressed in the superficial dorsal horn**. Light micrographs showing the distribution of neurons expressing Arc/Arg3.1 mRNA (***A, B***) or protein (***C***) in the rat spinal dorsal horn after peripheral stimulation with 25% mustard oil gauze wrapped around one hind paw for 2 hours. Note that many of the Arc/Arg3.1 labeled neurons are located in laminae I&II, and very few labeled neurons are present below lamina II. ***D*, **Fluorescent micrograph showing the expression of Arc/Arg3.1 mRNA and its colocalization with the neuronal marker NeuN. Arrowheads indicate Arc/Arg3.1 mRNA and NeuN double labeled neurons. Scale bar: 50 μm (***A ***and ***D***); 25 μm (***B***); 100 μm (***C***).

In the spinal cord of naïve rats and mice there was no detectable expression of Arc/Arg3.1 mRNA or protein when using in situ hybridization (ISH) and immunohistochemistry (IHC), respectively. However, after application of a peripheral nociceptive stimulus to the hind paw, Arc/Arg3.1 was expressed in a limited number of cells in the superficial layers of the lumbar dorsal horn. ISH using the alkaline phosphatase (AP) reaction produced a bluish/brownish reaction product in the cytoplasm and in some occasions in the nucleus and primary dendrites of Arc/Arg3.1 mRNA expressing neurons (Fig. 1A,B). Arc/Arg3.1 protein, visualized by bright field IHC, was present primarily in the cytoplasm, occasionally combined with nuclear labeling or labeling in proximal dendrites (Fig. 1C). Applying fluorescent IHC for Arc/Arg3.1 protein produced very similar labeling characteristics. In order to ascertain that Arc/Arg3.1 is expressed in neurons rather than in glial cells, we combined FISH for Arc/Arg3.1 mRNA with fluorescent IHC for NeuN, which is a specific marker for neuronal cells (Fig. 1D). It was found that 95% ± 1.3 (SEM) of the cells expressing Arc/Arg3.1 mRNA also expressed NeuN (99% ± 0.4 for 25% MO/1 h, n = 4; 95% ± 3.3 for 25% MO/2 hrs, n = 5; 94% ± 2.8 for CFA for 1.5 hrs, n = 4).

For both ISH and IHC, we observed that the intensity of the labeled neurons varied in a single section. We did not observe any labeling indicative of localization of Arc/Arg3.1 in intermediate or distal dendrites. Although labeling patterns obtained with ISH and IHC were identical, labeling efficiency was higher for ISH than for IHC. Therefore, ISH was used for the quantification of neurons expressing Arc/Arg3.1. The specificity of our detection techniques was assessed by omitting the probes/primary antibodies in the ISH and IHC procedures, respectively, and by applying ISH and IHC on spinal tissue of Arc/Arg3.1 KO mice. These experiments did not show any labeling in the spinal cord. ISH performed on cortex of naïve rats showed Arc/Arg3.1 mRNA labeling in the cortex and the hippocampus as previously reported [17].

### Distribution and quantification of Arc/Arg3.1 mRNA expressing neurons in the spinal cord following nociceptive stimulation

**Figure 2:**
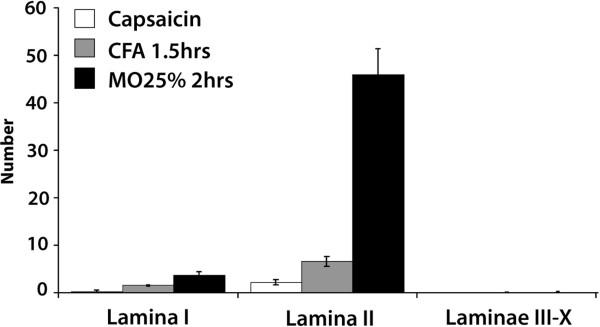
**Several types of nociceptive stimuli induce Arc/Arg3.1 mRNA in the ipsilateral superficial dorsal horn**. Histogram showing the number of Arc/Arg3.1 mRNA labeled neurons after each specific nociceptive stimulus. Error bars represent ± SEM.

Several types of nociceptive stimuli applied to the hind paw induced Arc/Arg3.1 mRNA expressing neurons on the ipsilateral side (Fig. 2) but not on the contralateral side of the lumbar superficial dorsal horn. A single subcutaneous injection of capsaicin resulted in the lowest average number of labeled neurons per section (2.6 ± 0.6 SEM, n = 6), while wrapping the paw in a gauze soaked with 25% mustard oil (MO) for 2 hrs induced the highest number of neurons (50 ± 3.5 SEM, n = 5). On average, lamina II accounted for 85% ± 3.5 of the labeled neurons, while lamina I (14% ± 3.2) and III (0.6% ± 0.4) contained the remaining labeled neurons. The other laminae very rarely contained labeled neurons.

### Expression of Arc/Arg3.1 mRNA following nociceptive stimulation occurs in a subset of c-Fos labeled neurons and is intensity dependent

**Figure 3:**
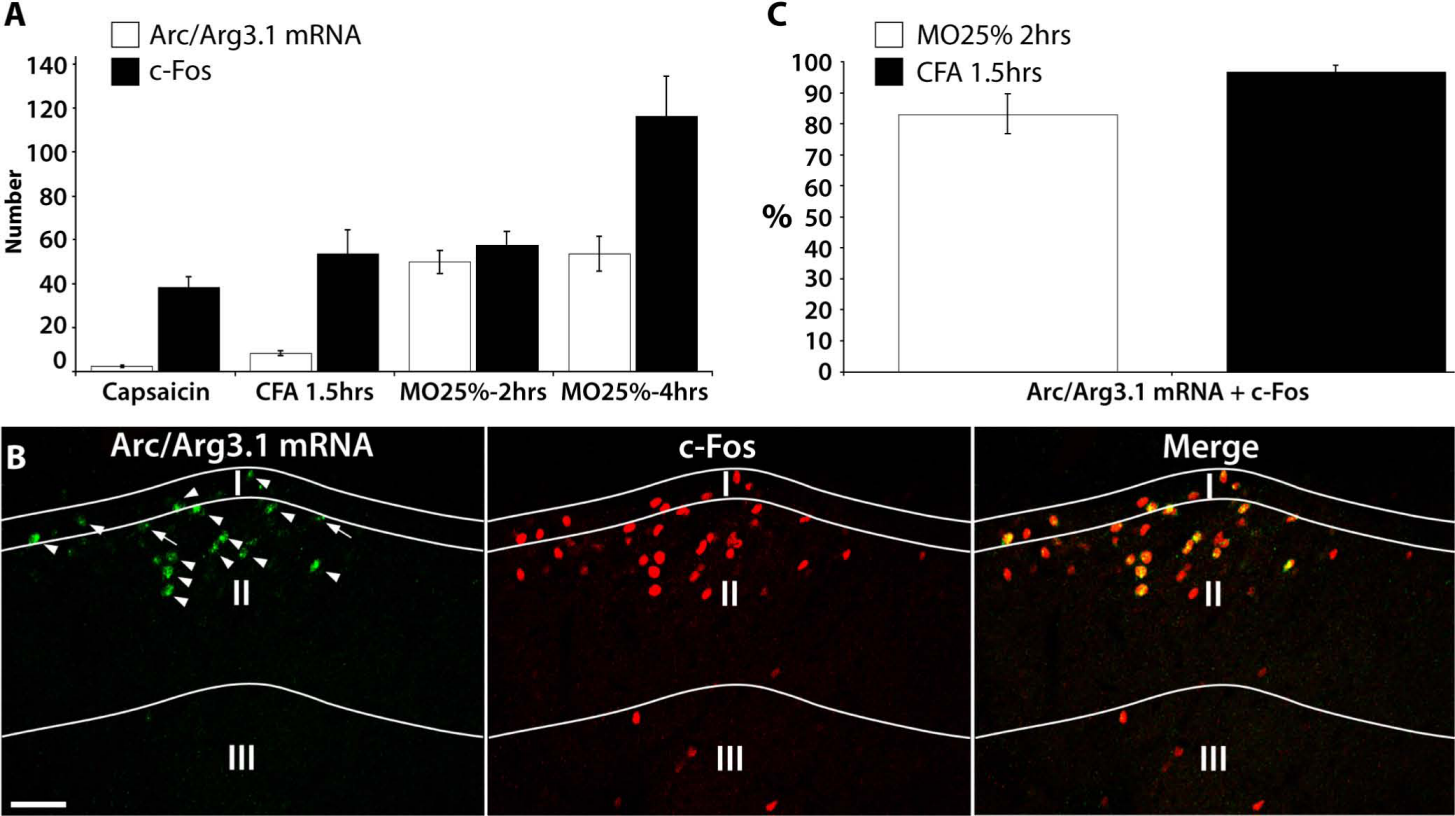
**The number of neurons expressing c-Fos outnumber Arc/Arg3.1 mRNA labeled neurons**. ***A***, Histogram showing the number of neurons labeled for c-Fos or Arc/Arg3.1 mRNA following various nociceptive stimuli. capsaicin, n = 6; CFA1.5 hrs, n = 4; MO25%-2 hrs, n = 4; MO25%-4 hrs, n = 4. ***B***, Fluorescent micrograph showing Arc/Arg3.1 mRNA and/or c-Fos protein labeled neurons in spinal dorsal horn after stimulation with 25% mustard oil for 2 hours. Arrows indicate Arc/Arg3.1 mRNA labeled neurons that were not in focus and therefore not included in the analysis. Arrow heads indicate Arc/Arg3.1 mRNA and c-Fos protein labeled neurons. ***C***, Histogram showing the percentage of Arc/Arg3.1 mRNA labeled neurons that also express the neuronal activation marker c-Fos. Scale bar: 50 μm. Error bars represent ± SEM.

**Figure 4:**
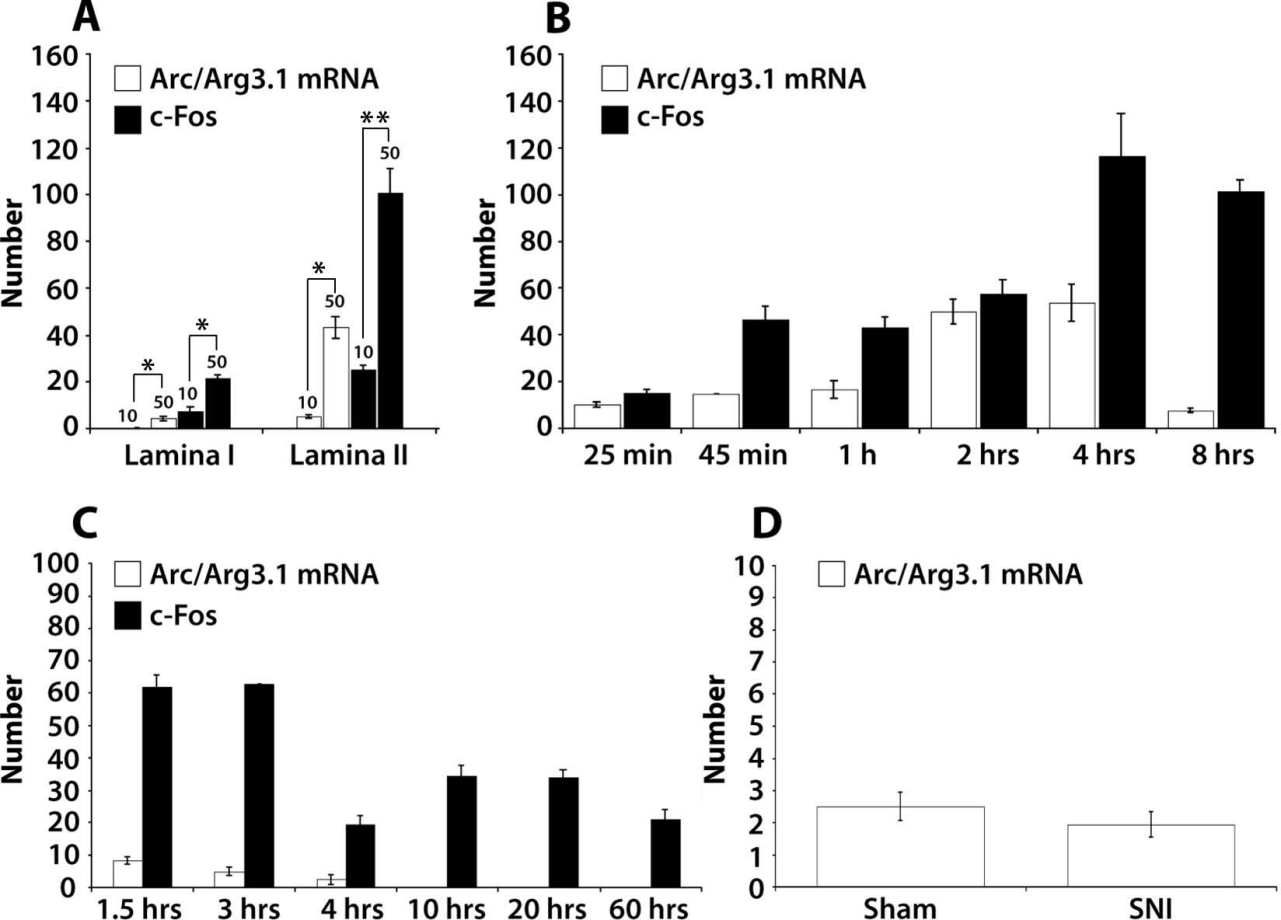
**Arc/Arg3.1 expression is stimulus intensity dependent and is present only in the acute phase of chronic inflammatory and neuropathic pain**. ***A*, **Histogram showing the numbers of Arc/Arg3.1 mRNA or c-Fos protein labeled neurons at 2 hours after a brush applied stimulation with either 10% (n = 5) or 50% (n = 5) mustard oil. * = *p *< 0.05; ** = *p *< 0.005 (unpaired *t*-test). ***B***, Time course of Arc/Arg3.1 mRNA and c-Fos protein expression after stimulation with 25% mustard oil gauze wrapped around one hind paw for different survival times. 25 min, n = 4; 45 min, n = 4; 1 h, n = 4; 2 hrs, n = 5; 4 hrs, n = 4; 8 hrs, n = 4. ***C***, Time course of Arc/Arg3.1 mRNA and c-Fos protein expression after CFA injection in the hind paw. 1.5 hrs, n = 4; 3 hrs, n = 4; 4 hrs, n = 4; 10 hrs, n = 4; 20 hrs, n = 4; 60 hrs, n = 4. ***D***, The number of Arc/Arg3.1 mRNA labeled neurons 2 hours after sham operation (n = 3) or after cutting the common peroneal and tibial nerves in the SNI model (n = 3). Error bars represent ± SEM.

The number of neurons expressing the neuronal activation marker c-Fos or Arc/Arg3.1 mRNA was counted in separate sections treated with IHC or ISH, respectively. c-Fos labeled neurons outnumbered Arc/Arg3.1 mRNA labeled neurons (Fig. 3A), except after 2 hrs mustard oil stimulation when about equal number of neurons were labeled. FISH and fluorescent IHC were applied to simultaneously visualize Arc/Arg3.1 mRNA and c-Fos protein, respectively (Fig. 3B). When data from the 25% mustard oil and the CFA groups were taken together (Fig. 3C), 90% ± 6.8 of the Arc/Arg3.1 mRNA expressing neurons also contained c-Fos protein. In order to determine whether the number of Arc/Arg3.1 expressing neurons was stimulus intensity dependent, rats received a single application (by brush) of either 10% (n = 5) or 50% (n = 5) mustard oil on one hind paw. It was found that 50% MO induced significantly higher numbers of Arc/Arg3.1 mRNA positive neurons that 10% mustard oil (Fig. 4A). The number of c-Fos labeled neurons showed a similar significant increase.

### Temporal expression of Arc/Arg3.1 mRNA in an acute and a chronic pain model

As a model for acute pain, 25% MO soaked gauze was wrapped around one hind paw, with survival times ranging from 25 min to 8 hrs. The number of Arc/Arg3.1 mRNA expressing neurons increased over time, reached a peak at 4 hours and then declined (Fig. 4B). The distribution of labeled neurons remained unchanged over time. As a model for chronic pain CFA was injected in the hind paw, with survival times ranging from 1.5 hrs to 60 hrs. Temporal expression of Arc/Arg3.1 mRNA was highest at 1.5 hrs post injection and then gradually declined (Fig. 4C). No expression of Arc/Arg3.1 mRNA was found at survival times of 10 hrs and longer. The number of c-Fos expressing neurons was increased at all survival times. In the spared nerve injury (SNI) model for neuropathic pain, expression of Arc/Arg3.1 mRNA was only observed at two hours after the operation. Arc/Arg3.1 mRNA was not expressed 1 week or 2 weeks after the operation (not shown) when the neuropathic pain symptoms, i.e. mechanical and thermal hyperalgesia and allodynia, had developed. There was no significant difference in the number of Arc/Arg3.1 mRNA labeled neurons between the SNI and sham operated group (*p*>0.05, unpaired *t*-test) (Fig. 4D).

### Arc/Arg3.1 mRNA is expressed in specific subpopulations of dorsal horn neurons

**Figure 5:**
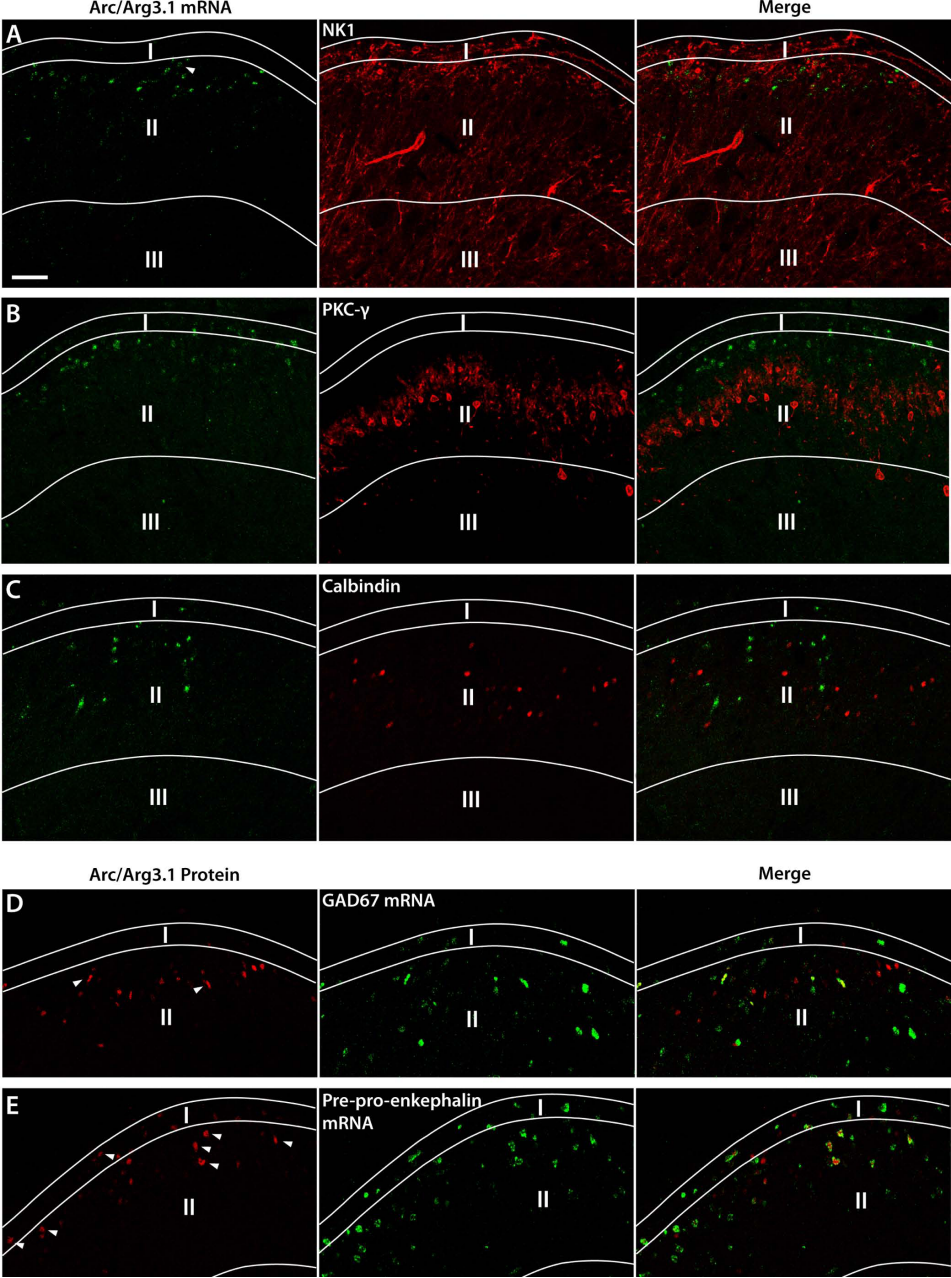
**Arc/Arg3.1 is expressed in a subpopulation of superficial dorsal horn neurons with a preference for neurons containing enkephalin**. ***A-E***, Fluorescent micrographs showing neurons in the superficial dorsal horn that express Arc/Arg3.1 mRNA (***A-C***) or protein (***D and E***) and markers that identify neurons expressing the neurokinin-1 receptor (NK1), protein kinase C gamma (PKC-γ), Calbindin, GAD67 mRNA (GABAergic neurons), or preproenkephalin mRNA (enkephalinergic neurons) respectively. The following nociceptive stimuli were used. ***A***, CFA, survival time 1.5 hrs, ***B and C***: Mustard oil 25% gauze wrapped, survival time 1 h, ***D and E***, Mustard oil 25% gauze wrapped, survival time 2 h. Arrow heads indicate Arc/Arg3.1 labeled neurons that also express one of the markers mentioned above. Scale bar: 50 μm.

**Figure 6:**
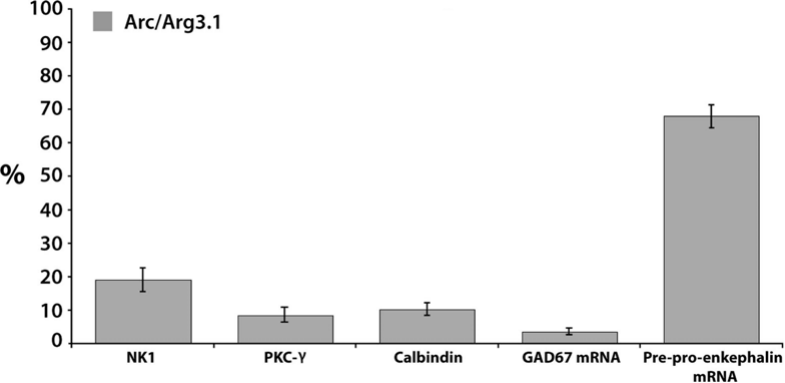
**Histogram showing the averaged percentages of neurons expressing Arc/Arg3.1(induced after various nociceptive stimuli, as in fig. 5), that co-express either NK1, PKC-γ, Calbindin, GAD67 mRNA or preproenkephalin mRNA**. Error bars represent ± SEM. For details, see text.

In this experiment, the colocalization of Arc/Arg3.1 with various neuronal markers was investigated (Fig. 5A-E). We found that about a fifth of Arc/Arg3.1 mRNA positive neurons also express the NK-1 receptor (CFA 1.5 hrs: 21.7% ± 7.6, n = 4; MO25%/2 hrs: 17% ± 3.4, n = 5) (Fig. 6). Less than 10% of Arc/Arg3.1 mRNA expressing neurons also expressed PKC-γ protein (CFA 1.5 hrs: 7.7% ± 3.7, n = 4; MO25%/1h: 9.3% ± 3.8, n = 4). Further, Arc/Arg3.1 mRNA expressing neurons showed a low level of co-existence with calbindin (CFA 1.5 hrs: 9.7% ± 1.4, n = 4; MO25%/1h: 10.5% ± 2.6, n = 4).

In order to identify Arc/Arg3.1 in inhibitory neurons, FISH for GAD67 mRNA, the specific marker for GABAergic neurons, and fluorescent IHC for Arc/Arg3.1 protein were combined. Very few of the Arc/Arg3.1 labeled neurons were GABAergic (CFA 3 hrs: 1.7% ± 0.8, n = 4; MO25%/2 hrs: 4.5% ± 0.8, n = 5; MO25%/4 hrs: 4.5% ± 1.5, n = 4) (Fig. 6). Preproenkephalin mRNA is a marker for the subpopulation of enkephalinergic neurons in the spinal cord. Interestingly, a large majority of the Arc/Arg3.1 positive neurons also expressed preproenkephalin mRNA (CFA 3 hrs: 74.2% ± 9.2, n = 4; MO25%/2 hrs: 61.5% ± 2.6, n = 4; MO25%/4 hrs: 68.1% ± 3, n = 4) (Fig. 6).

### Intrathecal injection of BDNF induces Arc/Arg3.1 mRNA expression

**Figure 7:**
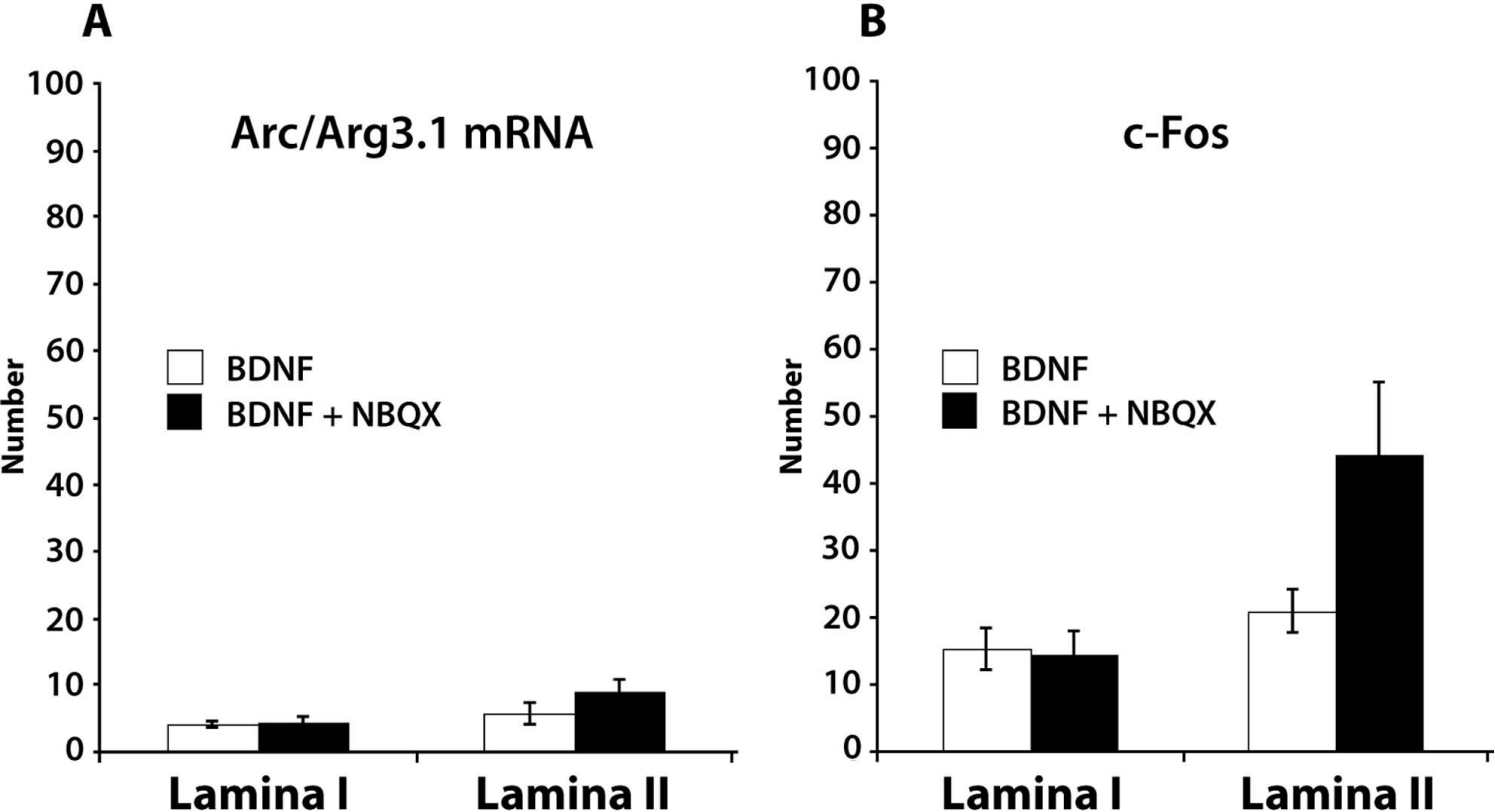
**Intrathecal injection of BDNF induces Arc/Arg3.1 mRNA expression in spinal dorsal horn**. ***A and B***, The averaged number of neurons that express Arc/Arg3.1 mRNA (***A***) or c-Fos protein (***B***) after intrathecal injection with BDNF or BDNF together with NBQX. Error bars represent ± SEM.

Intrathecal injection of brain-derived neurotrophic factor (BDNF) induced Arc/Arg3.1 mRNA expression in the superficial dorsal horn neurons (10 ± 1.7/section, n = 6). We found that 45% ± 8 of Arc/Arg3.1 mRNA labeled neurons were located in lamina I and 55% ± 8 in lamina II. 93.6% ± 2.5 of Arc/Arg3.1 mRNA labeled neurons expressed NeuN, 55.6% ± 9.1 expressed c-Fos, and 16.8% ± 6.4% expressed NK-1. Since it has been shown [18] that administration of BDNF together with NBQX, which is an AMPA receptor blocker, increases Arc/Arg3.1 mRNA expression in cortical neurons, we injected BDNF intrathecally together with NBQX. This combination resulted in 13.8 ± 2.9 Arc/Arg3.1 mRNA labeled neurons/section (n = 6) (Fig. 7A), which was not significantly different from intrathecal BDNF injection alone (unpaired *t*-test). c-Fos expression after BDNF + NBQX injection was also not significantly different from BDNF injection alone (*p*= 0.08 for lamina II) (Fig. 7B). Intrathecal injection of vehicle (n = 2) or NBQX (n = 2) alone did not induce Arc/Arg3.1 mRNA expression in the spinal cord. Furthermore, we found that intrathecal injection with NMDA (n = 2), which served as a positive control, also induced Arc/Arg3.1 expression in the dorsal horn (not shown).

### Pain behavior in the Arc/Arg3.1 KO mice

#### Mechanical and thermal thresholds

**Figure 8:**
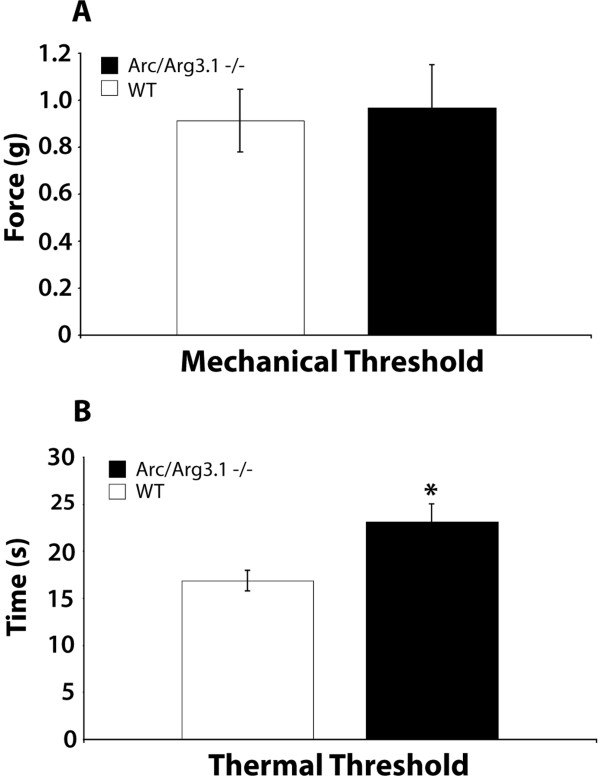
**Naïve Arc/Arg3.1 knockout (KO) mice showed significantly longer response times on the hotplate than their naïve wild type (WT) littermates**. ***A and B***, Histogram showing the mechanical threshold (***A***) and thermal withdrawal latency (***B***) of Arc/Arg3.1 KO and WT mice, assessed with the Von Frey and the hotplate test, respectively. Error bars represent ± SEM. *: *p *< 0.05, unpaired *t*-test; n = 4 for A and for B.

Freely moving Arc/Arg3.1 knockout (KO) mice did not display any overt behavioral abnormalities in comparison with their wild type (WT) littermates, as reported previously [13]. With respect to pain behavior, the mechanical thresholds and hot plate withdrawal latencies were tested. We found that the mechanical thresholds in Arc/Arg3.1 KO mice were not significantly different from their WT littermates (Fig. 8A). However, in the hotplate test Arc/Arg3.1 KO mice showed significantly longer withdrawal latencies than WT mice (Fig. 8B).

#### Acute pain: formalin test

**Figure 9:**
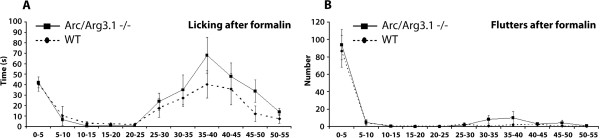
**Arc/Arg3.1 KO mice do not differ from WT mice concerning licking or fluttering of the paw injected with formalin**. ***A and B***, Graphs showing the time spent licking (***A***) and the number of flutters (***B***) after formalin injection in the hind paw during an observation period of 55 minutes. Error bars represent ± SEM. Differences were not significant (repeated-measures ANOVA, *p*>0.05). n = 4 for A and for B.

Subcutaneous injection of formalin in the hind paw induced a two-phased pain behavior in both WT and Arc/Arg3.1 KO mice, consisting of licking and fluttering of the injected paw. In both groups, the first phase was apparent in the first 10 minutes after injection, and the second phase began 25 minutes after injection with licking as the prominent behavior. No significant difference (repeated-measures ANOVA, *p*>0.05) was found between the WT and Arc/Arg3.1 KO mice in licking or fluttering behavior (Fig. 9A,B). Also the total licking time (WT: 200 sec. ± 34 (SEM); KO: 275 sec. ± 49 (SEM); *p*>0.05, unpaired *t*-test) nor the total numbers of flutters (WT 100 ± 21 (SEM); KO 128 ± 32 (SEM); *p*>0.05, unpaired *t*-test) were significantly different. In addition, c-Fos expression due to the formalin injection did not appear different from the c-Fos expression in the WT mice.

#### Chronic pain: inflammation

**Figure 10:**
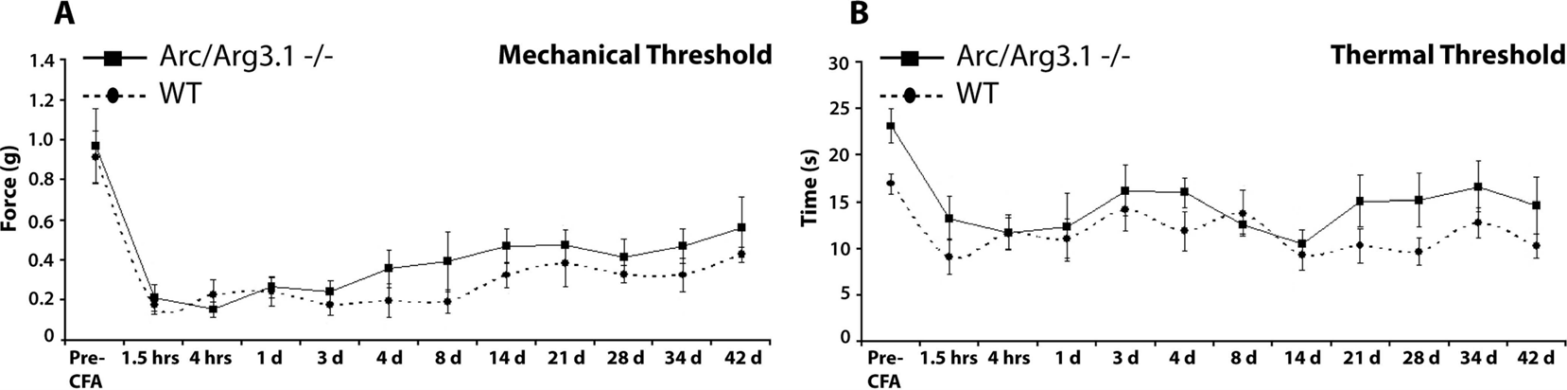
**No differences in mechanical and thermal thresholds between the Arc/Arg3.1 KO and WT mice during the time course of inflammatory pain**. ***A and B***, Graphs showing mechanical (***A***) and thermal (***B***) thresholds after CFA injection in one hind paw of Arc/Arg3.1 KO and WT mice. Differences were not significant at any time point (repeated-measures ANOVA, *p*>0.05). n = 4 for A and for B.

Induction of chronic inflammation by CFA injection in the hind paw resulted in decreased mechanical thresholds of the injected paw (Fig. 10A). A repeated measures ANOVA did not reveal any significant differences between WT and Arc/Arg3.1 KO mice regarding the mechanical or thermal thresholds at any time point (Fig. 10A,B).

## Discussion

In this study we have used in situ hybridization (ISH) and immunohistochemistry (IHC) to show that nociceptive stimulation induced Arc/Arg3.1 mRNA and protein in the superficial dorsal horn of the spinal cord. Both techniques specifically identified Arc/Arg3.1 since standard controls, most notably nociceptively stimulated spinal cord of Arc/Arg3.1 knockout (KO) mice, did not show any specific labeling. In naïve or vehicle treated animals expression of Arc/Arg3.1 mRNA and protein was absent in the spinal cord, in agreement with a study using RT-PCR [19]. This strongly indicates that in the spinal cord a nociceptive stimulus induces *de novo *expression of Arc/Arg3.1, in contrast with other areas of the nervous system, like hippocampus [17] and cortex [20].

Arc/Arg3.1 mRNA and protein were induced in the superficial dorsal horn in the acute phases of all pain models that we tested, i.e. after nociceptive stimulation with capsaicin, CFA, formalin and mustard oil. Injection of CFA induces an inflammatory process [21] that leads to the release of cytokines and other local messengers, all of which may activate different types of receptors on nociceptive fibers. Capsaicin, however, specifically activates nociceptive fibers expressing the transient receptor potential vanilloid-1 (TRPV1) [22]. Further, mustard oil and formalin both specifically activate the TRPA1 receptor, although formalin may exert TRPA1-independent effects at higher concentrations [23,24]. The number of neurons producing Arc/Arg3.1 mRNA varied in the different pain models, and increasing the intensity of the pain stimulus resulted in an increased number of neurons expressing Arc/Arg3.1 as shown in the mustard oil experiments. Therefore, our data indicate that the number of neurons expressing Arc/Arg3.1 depends on the intensity of the stimulus, but is not limited to the activation of one specific receptor on peripheral nerves.

Neurons expressing Arc/Arg3.1 in the spinal cord are most likely driven by direct input from afferent nociceptive fibers that use glutamate as their main neurotransmitter [25]. Apart from glutamate and various neuropeptides, these fibers may also contain growth factors like BDNF [26] or GDNF [27]. We found that intrathecal injection of NMDA or BDNF induced Arc/Arg3.1 mRNA in spinal dorsal horn neurons. This is in line with Arc/Arg3.1 expression in cultured neurons following BDNF application [18]. The same study showed a significantly enhanced expression of Arc/Arg3.1 mRNA when NBQX, a potent AMPA receptor blocker, was applied together with BDNF. However, in the present study a significant increase in the number of Arc/Arg3.1 mRNA expressing neurons could not be confirmed after intrathecal injection of BDNF and NBQX together. Taken together, our findings are in line with the idea that release of glutamate and/or BDNF from activated nociceptive fibers are at least partly responsible for Arc/Arg3.1 induction in the spinal dorsal horn.

Following nociceptive stimulation, Arc/Arg3.1 was often expressed in activated neurons as identified by c-Fos. Especially after nociceptive stimulation with capsaicin, and after chronic inflammatory pain, the number of neurons expressing Arc/Arg3.1 is low as compared to those showing c-Fos expression. This finding may be interpreted to indicate that Arc/Arg3.1 is only expressed in activated neurons that received the strongest input from nociceptive fibers. This assumption is in line with our finding that Arc/Arg3.1 expression is intensity dependent. On the other hand, there may be specific subpopulations of spinal nociceptive neurons that are capable of producing Arc/Arg3.1, while others are not. In search of such a neuronal subpopulation that specifically expressed Arc/Arg3.1, we focused on neurons that were characterized by the expression of the neurokinin-1 (NK-1) receptor, Protein Kinase C gamma (PKC-γ), calbindin, GAD67 or preproenkephalin. We found a high percentage of Arc/Arg3.1 expressing neurons (68%) to contain preproenkephalin, while percentages of colocalization with other markers were less prominent (19% for NK-1; 8.5% for PKC-γ; 3.6% for GAD67; 10% for calbindin). NK-1 expressing neurons project to supraspinal sites [28] and are essential for the initiation and maintenance of chronic neuropathic and inflammatory pain [29], and neurons expressing PKC-γ are considered critically important for the development of neuropathic pain after peripheral nerve injury [30]. The finding that only a small number of Arc/Arg3.1 positive neurons also expressed NK-1 or PKC-γ indicates that Arc/Arg3.1 is not strongly involved in pain processing by the NK-1 or PKC-γ subpopulations of dorsal horn neurons. This is remarkable since especially the NK-1 expressing neurons projecting to the parabrachial area or periaqueductal grey show LTP formation after high or low frequency stimulation, respectively [31]. Our finding indicates that Arc/Arg3.1 dependent long term changes may occur preferentially in local interneurons rather than in projection neurons. Further, we found low colocalization with GAD67, the marker for GABAergic neurons, indicating that the expression of Arc/Arg3.1 is low in the total subpopulation of dorsal horn inhibitory neurons since glycinergic neurons are virtually absent in the superficial dorsal horn [32-34], and, if present, also contain GABA [35]. In the hippocampal and neocortical neurons expression of Arc/Arg3.1 in GABAergic positive neurons is also low but this is not the case in the dorsal striatum [20]. Together, NK-1, PKC-γ and/or preproenkephalin constitute more than 90% of the Arc/Arg3.1 expressing neurons. Since to date there is no evidence for the colocalization of these substances with each other, we conclude that Arc/Arg3.1 is preferentially expressed in the subpopulation of enkephalinergic neurons. Preproenkephalin mRNA is the precursor of both Met- and Leu-enkephalin, which are both expressed by neurons in the spinal cord and mainly exert their effect on the δ-opioid receptor (DOR) [36]. Also, preproenkephalin mRNA in the spinal cord is increased after peripheral inflammation and is also present in neurons that express c-Fos after nociceptive stimuli [37]. Further, using VgluT2 immunohistochemistry for identifying glutamatergic terminals, it was shown [38] that 85% of the enkephalin containing terminals in the dorsal horn use glutamate as transmitter. However, a study [39] using cultured dorsal horn neurons showed 42% colocalization of immunohistochemically identified GAD and enkephalin. A more recent study [40] using preproenkephalin green fluorescent protein transgenic mice, showed that 43% of the fluorescent enkephalin neurons also expressed immunohistochemically identified GABA. Colocalization of enkephalin with VgluT2 was not explored in these studies. Since we found a low level of colocalization of Arc/Arg3.1 with GABAergic neurons, it is not unlikely that several of the enkephalinergic neurons in the spinal cord that express Arc/Arg3.1 also use glutamate as a transmitter. The functional role of glutamate in these fibers is unclear, since it is not known whether they activate inhibitory or excitatory (i.e. anti- or pro-nociceptive) circuits in the spinal cord, nor is it known under which circumstances enkephalin and/or glutamate is released from these fibers. Since the activation of the delta opioid receptor (DOR), through which enkephalin exerts its effect, decreases pain behavior during chronic peripheral inflammation [41], we tend to conclude that the overall effect of Arc/Arg3.1 expressing enkephalinergic neurons is anti-nociceptive.

In order to understand the functional role of Arc/Arg3.1 in enkephalinergic neurons at the behavioral level, we employed Arc/Arg3.1 KO mice and their WT littermates. The only significant difference between these mice was that in the hotplate test the thermal threshold of naïve Arc/Arg3.1 KO mice was significantly higher as compared to naïve WT mice. This finding is difficult to interpret since naïve WT mice, like their KO littermates, do not show Arc/Arg3.1 expression in the spinal cord. One explanation may be that there is a very low basal expression of Arc/Arg3.1 that we and others [19] were not able to detect, and that the permanent lack of Arc/Arg3.1 in the KO mice may have altered spinal processing of nociceptive thermal stimuli over time. Alternatively there may be supraspinal changes in nociceptive processing. After nociceptive stimuli, we did not find any difference in the pain behavior between the KO and WT mice in the formalin test and chronic inflammatory pain model. We therefore conclude that Arc/Arg3.1 KO mice do not show a clear phenotypic change that can be attributed to pain transmission in the spinal cord.

Several studies have shown that in hippocampus knockdown of Arc/Arg3.1 leads to enhanced LTP in the early phase but impaired consolidation of LTP and long term depression (LTD) in the late phase [13]. In the spinal cord, LTP is one of the major components of central sensitization [16], especially in lamina I projecting neurons [31]. LTP leads to enhanced responsiveness of spinal nociceptive neurons, which is important for maintenance of hyperalgesia and allodynia during acute and chronic pain. Our finding that Arc/Arg3.1 KO mice develop hypersensitivity in acute and chronic pain models in the same way as their WT littermates, suggests that the LTP formation that contributes to central sensitization and subsequent developing hyperalgesia is unaffected by the lack of Arc/Arg3.1. It seems therefore that Arc/Arg3.1 is not critically involved in LTP as occurring in the dorsal horn projection neurons, which in line with our result that few NK-1 positive neurons express Arc/Arg3.1.

The low number of spinal projection neurons that express Arc/Arg3.1 may be explained by the fact that, in contrast to other areas of the brain, structural long-term changes in the excitability of these spinal neurons are counterproductive if they persist after the healing process has been completed. Our finding that Arc/Arg3.1 is expressed predominantly in enkephalinergic neurons may suggest that in these neurons long term changes are actually consolidated. However, Arc/Arg3.1 KO mice that lack consolidation of long term changes show normal pain behavior. This would not exclude that enkephalinergic neurons, which have an inhibitory effect on pain transmission, may serve as an anti-nociceptive mechanism against strong nociceptive inputs that may occur in the future.

## Conclusions

Our data show that Arc/Arg3.1, which is critically involved in consolidating long term structural changes in the forebrain, is preferentially induced in spinal enkephalinergic neurons after nociceptive stimulation. This finding suggests that Arc/Arg3.1 dependent memory formation in spinal pain transmission is a predominant feature of neurons, which are anti-nociceptive rather than pro-nociceptive.

## Methods

### Animal experiments

In this study we used 99 male Wistar rats and 16 Arc/Arg3.1 KO mice and their wild type littermates.

#### Rats

50 μl of 0.3% capsaicin (Sigma-Aldrich) solution consisting of 80% saline, 10% Tween-80, and 10% ethanol 100% (n: 6; survival: 1.5 hrs) or 100 μl of Complete Freund's Adjuvant (CFA, Sigma-Aldrich; n: 24; survival: 1.5 hrs, 3 hrs, 4 hrs, 10 hrs, 20 hrs, 60 hrs) was injected in a hind paw under anesthesia with 2% isofluorane in 30%O_2_/70%N_2_O. In experiments applying mustard oil (MO) (Allylisothiocyanat, Merck) the animals were kept under anesthesia during entire survival time and subsequent perfusion. For 25% MO application (n: 25; survival: 25 min, 45 min, 1 h, 2 hrs, 4 hrs, 8 hrs) the left paw was shaved and wrapped in a gauze soaked with MO and then covered with foil. For application of 10% (n: 5; survival: 2 hrs) and 50% (n: 5, survival: 2 hrs) MO, the left paw was shaved and MO was applied once at the beginning of the experiment using a brush. For the experiments using intrathecal injections, the same protocol was used as described in [42]. Brain-derived neurotrophic factor (BDNF, 10 μg, Tocris) was injected intrathecally in a total injection volume of 40 μl (n: 6; survival: 75 min). In another experiment, 5 μg of 1,2,3,4-tetrahydro-6-nitro-2,3-dioxo-benzo[*f*]quinoxaline-7-sulfonamide (NBQX, Tocris) was injected concomitantly with BDNF (n: 6; survival: 75 min). For control intrathecal experiments, 25 nmol N-Methyl-d-asparate (NMDA; Sigma, St. Louis, MO; n: 2; survival: 75 min), or only vehicle (1% bovine serum albumin in 0.025 M phosphate buffer; n: 2; survival: 75 min) or only NBQX (n: 2; survival: 75 min) was injected intrathecally. After the injections, the rats were placed back in their cages. For induction of neuropathic pain, the spared nerve injury (SNI) model and a control operation were used [43]. In short, the sciatic nerve was exposed and the three branches were isolated. The tibial and the common peroneal branches were ligated and then cut while the sural nerve was left intact (n: 9; survival: 2 hrs, 7 days, 14 days). As a control, the sciatic nerve was only exposed and isolated (n:7; survival: 2 hrs, 7 days, 14 days).

#### Arc/Arg3.1 KO and WT mice

All mice were habituated for 5 days to the experimenter, the experiment room, and the transparent cage that was used for the Von Frey measurements. Thereafter, prior to each experiment the mice were habituated for 30 minutes to the room in which the behavioral experiments took place.

##### Von Frey experiment

before each Von Frey measurement, the mice were allowed to habituate to a transparent cage (15 cm × 15 cm with a gridded floor) for 10 minutes. We used calibrated von Frey filaments, which were applied for 2 seconds at 5 seconds interval, and the threshold was set at 3 evoked responses in a maximum of 5 applications.

##### Hotplate test

the thermal thresholds were assessed by measuring the time a mouse spent on the hotplate (51°C) before showing a response like fluttering or licking of the hind paw, or jumping. Immediately after a response or after maximally 45 seconds, the mouse was taken off the hotplate.

##### The formalin pain model

the mice were restrained by the experimenter and 15 μl of formalin, i.e. a freshly made solution of 4% paraformaldehyde (PFA) in phosphate buffer (PB), was injected subcutaneously in the left hind paw. The number of flutters and the time spent licking of the injected paw were measured during 55 minutes post-injection. After 90 minutes the mice were perfused and the tissue was processed as described below. n = 4 for Arc/Arg3.1 KO mice; n = 4 for WT littermates.

##### The CFA pain model

25 μl of CFA was injected in a hind paw of restrained mice and thereafter the mechanical and thermal thresholds were assessed at 1.5 h, 4 hrs, 1 d, 3 d, 4 d, 8 d, 14 d, 21 d, 28 d, 34 d, and 42 d post-injection. n = 4 for Arc/Arg3.1 KO mice; n = 4 for WT littermates.

##### Statistical analysis

An unpaired *t*-test or a repeated measures ANOVA was performed, p < 0.05 was taken as significant.

##### Examination of the Arc/Arg3.1 KO mice spinal tissue

After experiments the mice were sacrificed and further processed for immunohistochemistry (IHC) or in situ hybridization (ISH). Histological examination of Arc/Arg3.1 KO mice spinal cord did not reveal any morphological abnormalities in comparison with their WT littermates.

### Tissue preparation

At the end of the survival times the animals received an overdose of sodium pentobarbital and were transcardially perfused with 100 ml saline (rats) or 10 ml (mice) followed by 750 ml of 4% PFA (rats) or 50 ml (mice) dissolved in 0.12 M phosphate buffer (PB), pH 7.4. The spinal cord was dissected and left overnight in a solution of 4% PFA and 30% sucrose at 4°C. Subsequently, sections were cut (30 μm) on a freezing microtome and collected in RNAse-free PB. Serial sections were cut and collected in 9 separate jars, and therefore sections in one jar were at least 270 μm apart. The sections were kept in a solution of 40% glycerol, 40% ethyleenglycol and 20% RNAse-free PB for long-term storage at -20°C.

### In situ hybridization and immunohistochemistry

The partial cDNA templates encoding the following mRNAs were used: Arc/Arg3.1 (3.5 kb, full length probe encoding the mus musculus Arc/Arg3.1 gene, GeneID: 11838; Image Clone number: 3498057), GAD67 (3.2 kb; a generous gift from Dr. A.J. Tobin, UCLA), preproenkephalin (0.95 kb, a generous gift from Dr. S.L. Sabol, NIH). The riboprobes were obtained by linearizing the recombinant plasmids with the appropriate restriction enzymes and RNA polymerases. The transcription was performed in the presence of digoxigenin (DIG)- or fluorescein-labeled 11-UTP (Roche). ISH based on alkalic phosphatase (AP) reaction was performed following the protocol described previously [32]. For fluorescent in situ hybridization (FISH) the following modifications were applied to the protocol. After riboprobe hybridization, the detection of DIG or fluorescein was achieved with sheep polyclonal anti-Dig antibody (Roche) or mouse monoclonal anti-fluorescein antibody (Roche), respectively (1:500; 48 hours at 4°C in phosphate buffer saline (PBS), 2% milk powder and 0.5% Triton X-100). Thereafter, the anti-DIG or anti-fluorescein primary antibodies were detected using biotinylated rabbit-anti-goat (Vector) or goat-anti-mouse (Vector), respectively. Subsequently, the sections were incubated with Avidin-Biotin-Complex (ABC, Vector) tagged with horseradish peroxidase (HRP). A tyramide amplification procedure was performed by reacting HRP with H_2_O_2 _and a self made FITC tyramide according to protocol described in [44]. Thereafter, the sections were washed in PBS and processed for fluorescent IHC using the following antibodies diluted in 2% milk power solution: rabbit anti-Arc (1/3000; a generous gift from Dr. D. Kuhl), rabbit anti-c-Fos (1: 40.000; Oncogene Research Products, La Jolla, CA), rabbit anti-neurokinin-1 (NK1; 1:5000; Advanced Targeting System, CA, USA), rabbit anti-calbindin (1:7000; Swant, Switzerland), rabbit anti-PKC-γ (1/750; Santa Cruz), and mouse anti-neuronal nuclei (NeuN) monoclonal antibody (1:5000, Chemicon). These primary antibodies were detected with Cy3 tagged fluorescent secondary antibodies donkey-anti-rabbit or donkey-anti-mouse (1:200). Thereafter, the section were washed in PB and mounted on slides and coverslipped with Vectashield (Vector).

### Data analysis

Analysis was carried out on L4 and L5 segments of the spinal cord, except for the BDNF experiments, in which also S1 and S2 segments were included in the analysis. Slides were systematically examined starting from the first section in the first row for the appropriate segmental level. The first 5 to 6 sections that were encountered and were not damaged during the procedure were included in the analysis [42]. For illustrations, light micrographs were made with a digital camera and confocal images with a Zeis LSM 510 confocal laser scanning microscope and a 20× objective. The images were processed using Adobe Photoshop and were not manipulated, except for brightness and contrast. Quantitative analysis of Arc/Arg3.1 mRNA positive neurons based on AP-ISH was achieved using a camera lucida microscope (Neurolucida, Microbrightfield Inc., Williston, VT). The grey and white matter and the boundaries between the laminae were drawn according to [45] and labeled neurons were identified only if the largest diameter was at least 10 μm, and the cell soma contained a bluish/brownish product. Labeled neurons were expressed as the average number of labeled neurons per section.

For double labeling based on FISH combined with fluorescent IHC, confocal images were analyzed using the Zeis LSM image browser. For each section, the dorsal horn showing Arc/Arg3.1 labeled neurons was analyzed in a vertical plane consisting of 9 slices with an optical thickness of 2.46-2.76 μm. Every fifth section was analyzed for double labeled neurons. For markers that label the cytoplasm, the criterion was that the diameter of a profile was at least 10 μm to be counted as a neuron. For statistical analysis, an unpaired *t*-test was performed, and *p *< 0.05 considered significant.

## Competing interests

The authors declare that they have no competing interests

## Authors' contributions

MH performed or contributed to all experiments, analyzed data and drafted the paper. JLMJ contributed to experiments and analysis. KB contributed to experiments. DK provided KO mice and gave advice. JCH conceived and supervised the project and edited the manuscript. All authors read and approved the final manuscript.
